# C-terminal and full length TDP-43 specie differ according to FTLD-TDP lesion type but not genetic mutation

**DOI:** 10.1186/s40478-019-0755-x

**Published:** 2019-07-02

**Authors:** Keith A. Josephs, Yong-Jie Zhang, Matthew Baker, Rosa Rademakers, Leonard Petrucelli, Dennis W. Dickson

**Affiliations:** 1 Department of Neurology, Divisions of Behavioral Neurology & Movement Disorders, Mayo Clinic, Rochester, MN USA; 2 Department of Neuroscience, Mayo Clinic, Jacksonville, FL USA

## Abstract

The transactive response DNA binding protein of 43 kDa (TDP-43) is an intranuclear protein involved in RNA splicing. Abnormally deposited TDP-43 is found in the brains of patients with frontotemporal lobar degeneration (FTLD). Different morphological characteristics of TDP-43 immunoreactive inclusions define the different variants of FTLD-TDP. Little is known about the relationships between TDP-43 specie (phosphorylated TDP-43, C-terminal fragments and full length TDP-43) and lesion types. Using novel antibodies that recognize phosphorylated TDP-43 (pTDP-43), a neoepitope in the C-terminal fragment of TDP-43 (cTDP-43) and the N-terminal, i.e. full length (nTDP-43) we assess the relative burden of pTDP-43, cTDP-43 and nTDP-43 in 8 different lesion types across FTLD-TDP type A-C. These include neuronal cytoplasmic inclusions, dystrophic neurites, neuronal intranuclear inclusions, fine neurites of the hippocampus, peri-vascular inclusions, Pick body-like inclusions, long thick dystrophic neurites and granular pre-inclusions. We also assess for associations with *progranulin (GRN)* and *C9ORF72* genetic mutations. For all eight lesion types, the highest burden was observed for pTDP-43. In six of the eight lesions studied, cTDP-43 burden was greater than nTDP-43 burden. However, we observed a higher burden of nTDP-43 to cTDP-43 for pre-inclusions. We also noted an equal-to-slightly higher burden of nTDP-43 to cTDP-43 for peri-vascular inclusions. There was not strong evidence for associations to be driven by mutation status although for neuronal cytoplasmic inclusions and dystrophic neurites *GRN*+ cases had higher burden of pTDP-43, cTDP-43 and nTDP-43 compared to *GRN*- cases, with nTDP-43 inclusions only observed in *GRN*+ cases. In addition, for pre-inclusions, cTDP-43 and nTDP-43 burden tended to be higher in *C9ORF72*- cases compared to *C9ORF72*+ cases, although this was not the case for pTDP-43. There is clear evidence that phosphorylation and C terminal fragments play an important role in lesion formation in FTLD-TDP. However, for some inclusions, particularly pre-inclusions, full-length TDP-43 appears to be playing a role.

## Introduction

The TAR DNA binding protein of 43 kDa (TDP-43) is a nuclear protein that is involved with RNA splicing and transcription repression [5, 6]. TDP-43 is present in the brains of individuals with frontotemporal lobar degeneration (FTLD-TDP) with or without concomitant motor neuron disease (MND) [1, 33]. It is known that after translation, TDP-43 undergoes a number of post-translational modifications, such as phosphorylation, ubiquitination and even proteolytic cleavage into abnormal C-terminal specie [1, 33, 46]. Phosphorylated TDP-43 is a characteristic feature of the inclusions in FTLD-TDP and is considered important in the pathogenesis [17]. It has also been shown that inclusions in the brains of cases of FTLD-TDP are characterized by the presence of C-terminal TDP-43 specie, but not full length TDP-43, suggesting that full length TDP-43 may not be a pathologic TDP-43 species in the brains of FTLD-TDP cases [21]. Frontotemporal lobar degeneration with TDP, however, is not a homogeneous entity in terms of morphology of the lesions (lesion type). Over the past decade, many different TDP-43 immunoreactive lesion types have been identified. Lesion types are important, and in fact when combined with the distribution of the lesions defines five different FTLD-TDP variants (A-E) [25, 27, 30, 38]. FTLD-TDP variants A-C are common, accounting for more than 95% of all FTLD-TDP [24], while variants D and E are rare [16, 27]. Little is known regarding differences in phosphorylation specie, C-terminal specie and full length TDP-43 by lesion type. In addition, there is heterogeneity in mutation status in FTLD-TDP with some cases being association with mutations in either the progranulin (*GRN*) gene [2, 12] or with mutations in the *C9ORF72* gene [13, 36] while other cases are not associated with either mutation. A further complicating factor is that there are associations between mutation status and lesion type. For example, *GRN* mutations are associated with FTLD-TDP type A and hence also neuronal intranuclear inclusions (NII) [16, 23] while *C9ORF72* mutations are associated with FTLD-TDP type B and hence also granular pre-inclusions [19, 36, 37]. As with lesion type, little is known about the association between mutation status and TDP-43 specie.

In this study, our primary aim was to determine whether there are any differences in the burden of phosphorylated TDP-43 specie, C-terminal specie and full length TDP-43 across the different lesion types. We also aimed to determine whether there is any evidence for any association with mutation status. We hypothesize that there would be differences across lesion types, as well as evidence for associations with mutation status.

## Materials and methods

### Case selection

The database of the brain bank at Mayo Clinic, Jacksonville, Florida was queried to identify a random selection of 24 cases received between 1997 to 2013 with a neuropathologic diagnosis of FTLD-TDP type A (*n* = 8), FTLD-TDP type B (*n* = 8) and FTLD-TDP type C (*N* = 8) in which slides were available for review and paraffin blocks were available for additional analyses for this study. All diagnoses were made by one expert neuropathologist (DWD). A diagnosis of FTLD-TDP and types were based on current consensus recommendations [25, 30]. Type A was diagnosed when there were neuronal cytoplasmic inclusions (NCIs) and dystrophic neurites (DNs), as well as NIIs. Type B was diagnosed when there were a predominance of NCIs with minimal DNs and no NIIs. Type C was diagnosed when there were abundant long thick neurites with minimal NCIs in the neocortex and no NIIs.

### Neuropathologic methods

All cases had undergone neuropathologic assessment by a single neuropathologist (DWD), and had standardized tissue sampling and semi-quantitation of Alzheimer’s disease pathology. Thioflavin-S fluorescent microscopy was used for the evaluation of the distribution of senile plaques and neurofibrillary tangles (NFT) which was then used to determine the Thal β-amyloid phase as previously described [32], and the Braak NFT stage [3]. Immunohistochemistry was also performed on all cases with a α-synuclein antibody (NACP, 1:3000 rabbit polyclonal, Mayo Clinic antibody) and with a phosphorylated tau antibody (PHF-1, 1:1000 mouse monoclonal, gift from Dr. Peter Davies). HpScl was assessed on hematoxylin-eosin stained sections and was diagnosed when neuronal loss and gliosis was identified in the CA1 sector and/or subiculum of the hippocampus [14].

### TDP-43 immunohistochemistry & semi-quantitation

**Fig. 1:**
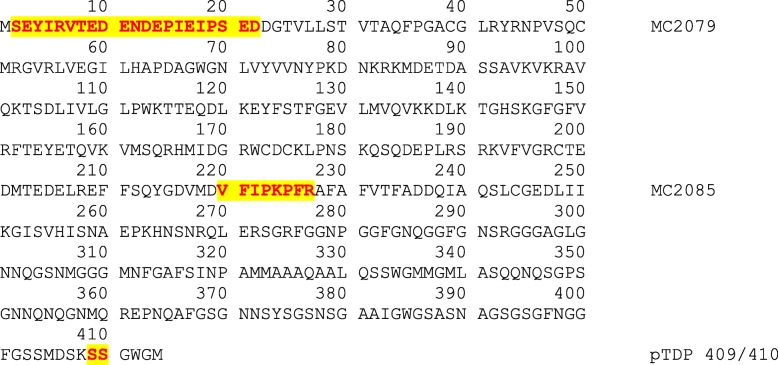
The amino acid sequence of TDP-43 from N terminus (1) to C terminus (414). The highlighted regions are the peptides used to generate the polyclonal antibodies (MC2079, MC2085 and pTDP 409/410)

In all 24 cases we performed serial sectioning and TDP-43 immunohistochemistry with four different TDP-43 antibodies: phosphorylated TDP-43 (pTDP-43) antibody (pS409/410, 1:5000 mouse monoclonal, Cosmo Bio Co., LTD) that recognizes TDP-43 with phosphorylated epitopes; anti TDP-43 antibody that recognizes a neoepitope in the C terminal fragment of cleaved TDP-43(cTDP-43) (MC2085, 1:2500 rabbit polyclonal, gift from Leonard Petrucelli, Mayo Clinic) [46]; anti TDP-43 antibody that recognizes an epitope in the amino terminus (nTDP-43) (MC1079; 1:2500 rabbit polyclonal, gift from Leonard Petrucelli, Mayo Clinic) [46] and a rabbit polyclonal anti-TDP-43 antibody (ProteinTech Group; Chicago IL; dilution 1:3000) which is a commercially available antibody. Antigens for the three non-commercial antibodies are shown in Fig. 1 and detailed biochemistry regarding these three antibodies have been previously published [7, 9, 10, 46]. Antibodies targeting phosphoserine 409 and 410 have been shown to have very strong immunoreactivity to the inclusions in FTLD-TDP [17, 22]. N-terminal TDP-43 fragments are believed to be rapidly degraded [4, 35] and as expected MC1079 binding represents detection of full length TDP-43 [46]. In all cases, immunohistochemistry was performed using a DAKO Autostainer (Universal Staining System Carpinteria, California). For this study, two investigators (KAJ & DWD) reviewed all 24 cases together and performed semi-quantitation of TDP-43 pathology with the three non-commercial antibodies (pTDP-43, cTDP-43 & nTDP-43) in the entorhinal cortex, CA1 sector, subiculum and dentate nucleus of the hippocampus. Lesions were considered positive, independent of the intensity of lesion staining. Hence, lesions showing slight/mild staining with one antibody were given equal weight to lesions with robust intensity with another antibody and both were assumed positive. This accounts for differences in affinities of the three different antibodies. For each region TDP-43 deposition was assessed independently for eight different types of lesions: (1) small discrete NCIs; (2) short/comma-like DNs; (3) fine neurites in the pyramidal layer of the hippocampus as described by Hatanpaa et al. [18]; (4) perivascular inclusions (typically bilobular and located next to capillary basal lamina) described by Lin et al. [28]; (5) Pick-body like inclusions in the dentate nucleus in FTLD-TDP type C cases as described by Katsuse and Dickson [26] (6) long thick DNs in cortex in FTLD-TDP type C cases, (7) granular pre-inclusions as described by Katsuse and Dickson [26], and (8) NIIs. These lesions were selected given that all have been associated with FTLD-TDP types. The burden of seven of the eight lesions was semi-quantitated on a 5-point scale in order to be able to compare across lesion type: 0 = no inclusions identified, 0.5 = scant number of inclusions; 1 = few inclusions, 2 = moderate inclusions and 3 = marked/frequent number of inclusions. We documented the presence/absence of NIIs given how rarely we identify this inclusion. For each lesion we assessed the burden detected with pTDP-43, cTDP-43 and nTDP-43.

### Genetic screening

In all 24 cases we screened for the presence of a mutation in the *GRN* and *C9ORF72* genes. Briefly, amplification by polymerase chain reaction (PCR) of exons 0-12 and the 3′ untranslated region of the *GRN* gene was performed using primers and protocols that have been previously described [2]. To assess for the presence of an expanded GGGGCC hexanucleotide repeat in *C9ORF72*, the repeat primed PCR was also used, as outlined in a previous publication [13].

## Results

### Demographic and pathological features by FTLD-TDP type

Demographic characteristics of the 24 cases are shown in Table 1. FTLD-TDP type A cases were mainly men, while type B cases were mainly women and type C cases were evenly split among men and women. FTLD-TDP type B cases were on average 7–9 years younger than type A and type C cases. The most common clinical diagnosis in the FTLD-TDP type A cases was behavioral variant frontotemporal dementia (bvFTD), while the most common clinical diagnosis for FTLD-TDP type B was FTD-MND and for type C was primary progressive aphasia (semantic variant). Of the eight FTLD-TDP type A cases, four had a mutation in the *GRN* gene and one had a mutation in the *C9ORF72* gene. Of the eight FTLD-TDP type B cases, four had a mutation in the *C9ORF72* gene. The Thal β-amyloid phase and the Braak NFT stage were both low and relatively similar across FTLD-TDP types; none were Braak NFT stage IV or greater and none met intermediate - high probability criteria for Alzheimer’s disease [43]. Hippocampal sclerosis was observed in all FTLD-TDP type A cases, but less than 25% of type B and C cases. All FTLD-TDP type B cases and two FTLD-TDP type A cases had pathological evidence of MND such as bunina bodies, neuronal loss and gliosis and TDP-43 immunoreactive inclusions in hypoglossal nucleus and/or loss of Betz cells from lamina VI of Broadman area 4. None of the FTLD-TDP type C cases had MND.

**Table 1:** Demographics and pathologic features of FTLD-TDP cases

	FTLD-TDP type A (*n* = 8)	FTLD-TDP type B (*n* = 8)	FTLD-TDP type C (*n* = 8)
Demographics			
Female Sex %	1 (12.5%)	5 (62.5%)	4 (50.0%)
Age at death	72 (60, 78)	63 (55, 79)	70 (65, 83)
Clinical diagnosis			
bvFTD	3 (37.5%)	3 (37.5%)	1 (12.5%)
PPA	1 (12.5%)	0	7 (87.5%)
FTD-MND/ALS	1 (12.5%)	5 (62.5%)	0
Other	3 (37.5%)	0	0
Genetic findings			
*C9ORF72*	1 (12.5%)	4 (50.0%)	0
*GRN*	4 (50.0%)	0	0
Co-pathologies			
Hippocampal sclerosis	8 (100%)	2 (25%)	1 (12.5%)
Braak NFT stage	1.0 (0.0, 2.0)	2.0 (1.0, 3.0)	1.5 (0.0, 3.0)
Thal Phases	0.5 (0.0, 3.0)	0.0 (0.0, 4.0)	0.5 (0.0, 2.0)
Pathological results			
*ERC/CA1/Sub*			
Phosphorylated TDP-43 (pTDP-43)	2.0 (1.0, 3.0)	2.5 (1.0, 3.0)	3.0 (1.0, 3.0)
C-Terminal specie TDP-43 (cTDP-43)	1.5 (0.5, 3.0)	0.5 (0.0, 3.0)	3.0 (0.5, 3.0)
Full length TDP-43 (nTDP-43)	1.0 (0.5, 2.0)	2.0 (0.0, 3.0)	2.0 (0.0, 3.0)
*Dentate nucleus of hippocampus*			
Phosphorylated TDP-43 (pTDP-43)	2.5 (1.0, 3.0)	3.0 (2.0, 4.0)	3.0 (2.0, 4.0)
C-Terminal specie TDP-43 (cTDP-43)	2.0 (1.0, 3.0)	2.0 (1.0, 4.0)	3.0 (1.0, 4.0)
Full length TDP-43 (nTDP-43)	1.0 (0.0, 1.0)	1.0 (0.0, 4.0)	1.0 (0.0, 3.0)

Data shown as median (range) and percentages

*bvFTD* behavioral variant frontotemporal dementia, *FTD-MND/ALS* Frontotemporal dementia with motor neuron disease/amyotrophic lateral sclerosis, *PPA* Primary progressive aphasia, *GRN* proganulin, *NFT* neurofibrillary tangle

There were no observable differences in lesion burden between the entorhinal cortex, CA1 sector and subiculum of the hippocampus, and hence these regions were combined for further analyses. In all instances the pTDP-43 antibody detected the greatest number of inclusions compared to cTDP-43 and nTDP-43 (Table 1). There were three cases (13%) where the pTDP-43 antibody detected lesions but neither cTDP-43 or nTDP-43 antibodies detected any lesions. This was observed for fine neurites, perivascular inclusions, NCI’s, DNs and pre-inclusions. We did not observe this phenomenon for long-thick dystrophic neurites. In all instances, except for in the entorhinal/CA1/subiculum in FTLD-TDP type B cases, the cTDP-43 antibody recognized a higher burden of lesions compared to the nTDP-43 antibody (Table 1). For the entorhinal/CA1/subiculum in FTLD-TDP type B cases we instead noted that the nTDP-43 antibody detected more inclusions compared to the cTDP-43 antibody.

### Associations of TDP-43 specie with lesion type

**Fig. 2:**
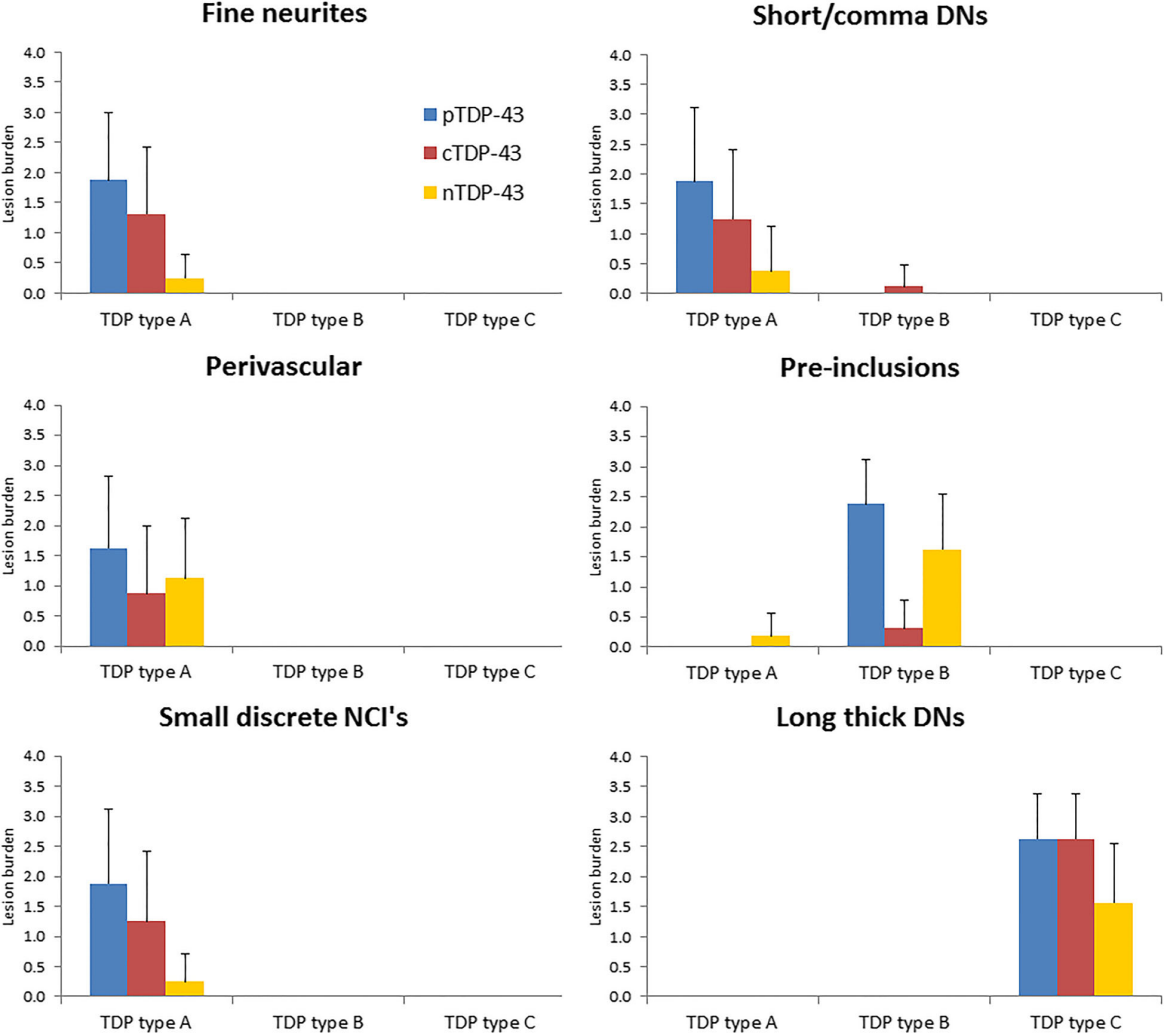
Histograms showing lesion burden detected using pTDP-43, cTDP-43 and nTDP-43 antibodies across FTLD-TDP types for the entorhinal cortex/CA1/subiculum. Lesion burden is based on semi-quantitation. The boxes represent mean burden with whiskers representing standard deviation

**Fig. 3:**
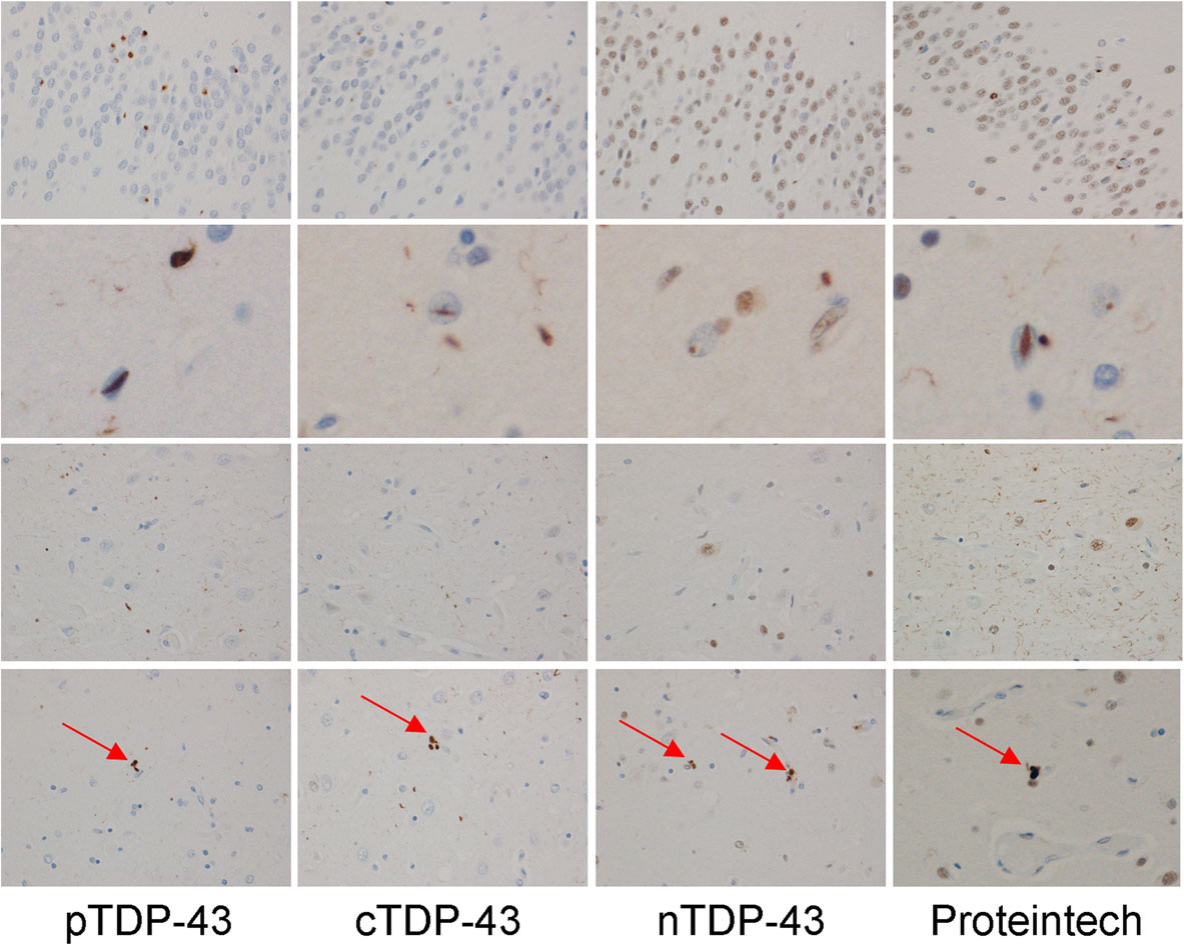
Detection of lesions in FTLD-TDP type A using pTDP-43, cTDP-43, nTDP-43 and the Proteintech antibodies. Top row shows dentate nucleus of the hippocampus, second row shows intranuclear inclusions in entorhinal cortex, third row shows CA1 and fourth row shows subiculum. Arrows depicts perivascular inclusions. Magnification × 20. Note the amount of background staining with the Proteintech antibody which prevents semi-quantitative analysis using this antibody

**Fig. 4:**
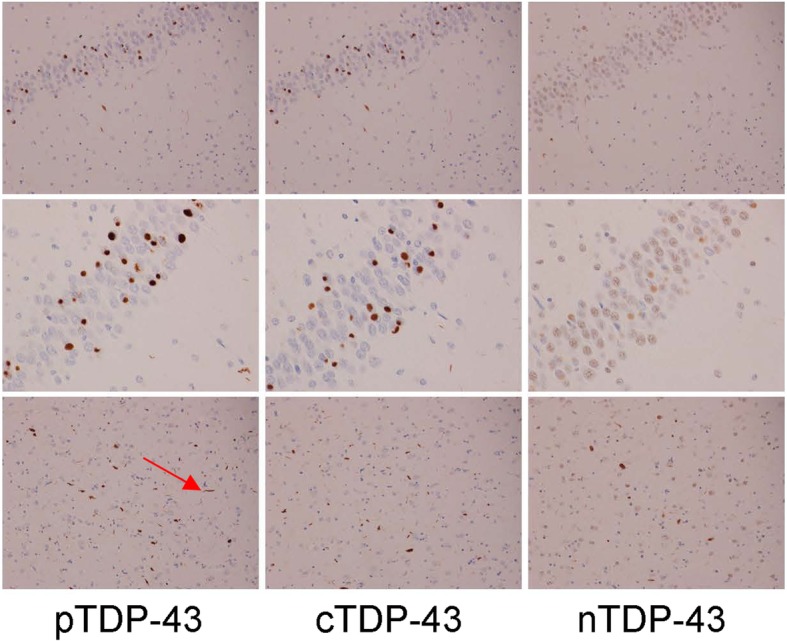
Detection of lesions in FTLD-TDP type C using pTDP-43, cTDP-43 and nTDP-43 antibodies. Top two rows show dentate nucleus of the hippocampus and bottom row shows entorhinal cortex. Arrow depicts long thick dystrophic neurites. Magnification × 10 for top row, and × 20 for bottom two rows

**Fig. 5:**
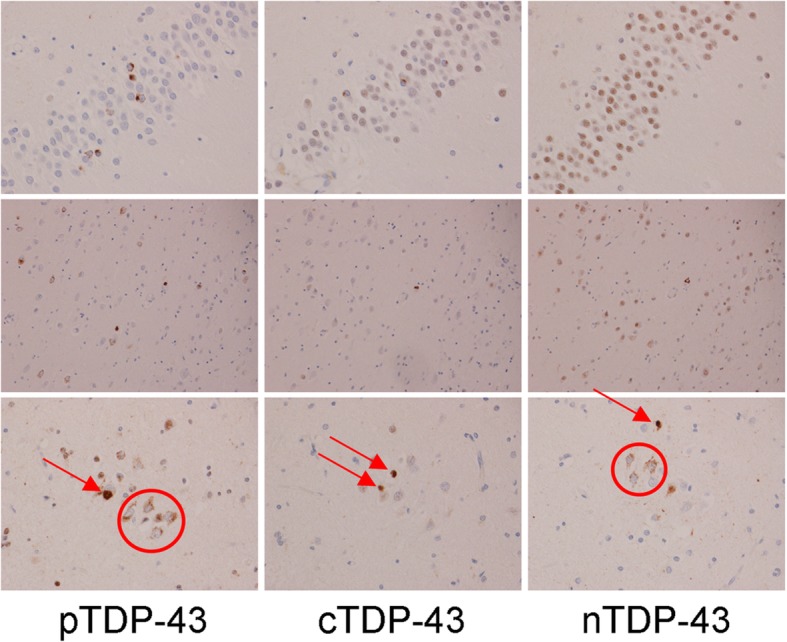
Detection of lesions in FTLD-TDP type B using pTDP-43, cTDP-43 and nTDP-43 antibodies. Top row shows dentate nucleus of the hippocampus and bottom two rows show entorhinal cortex with magnification × 10 for top row and × 20 for bottom row. Red circles depict pre-inclusions while arrows depict small discrete NCIs

In order to better understand the findings regarding the burden of pTDP-43, cTDP-43 and nTDP-43 that we observed, and to allow us to see whether the findings may be driven by any one or more lesion type, we compared pTDP-43, cTDP-43 and nTDP-43 burden for each of the six lesion types. In the entorhinal/CA1/subiculum (Fig. 2), we observed that for most lesion types there was a similar pattern of pTDP-43: cTDP-43: nTDP-43 burden. That is, we saw the greatest burden of inclusions to be detected by the pTDP-43 antibody. We also observed a greater burden of inclusions to be detected with the cTDP-43 antibody compared to the nTDP-43 antibody (Figs. 3 & 4). However, we noticed that pre-inclusions, which are typically observed in FTLD-TDP type B cases, showed a greater burden of inclusions detected with the nTDP-43 antibody compared to the cTDP-43 antibody (Fig. 5). We also noted a slightly higher burden of perivascular inclusions that were detected by the nTDP-43 antibody compared to the cTDP-43 antibody. Neuronal intranuclear inclusions (NIIs) were observed in five of the eight FTLD-TDP type A cases with the pTDP-43 antibody (data not shown). Of these five cases, NIIs were also observed in three cases with the cTDP-43 antibody. We did not detect NIIs with the nTDP-43 antibody.

**Fig. 6:**
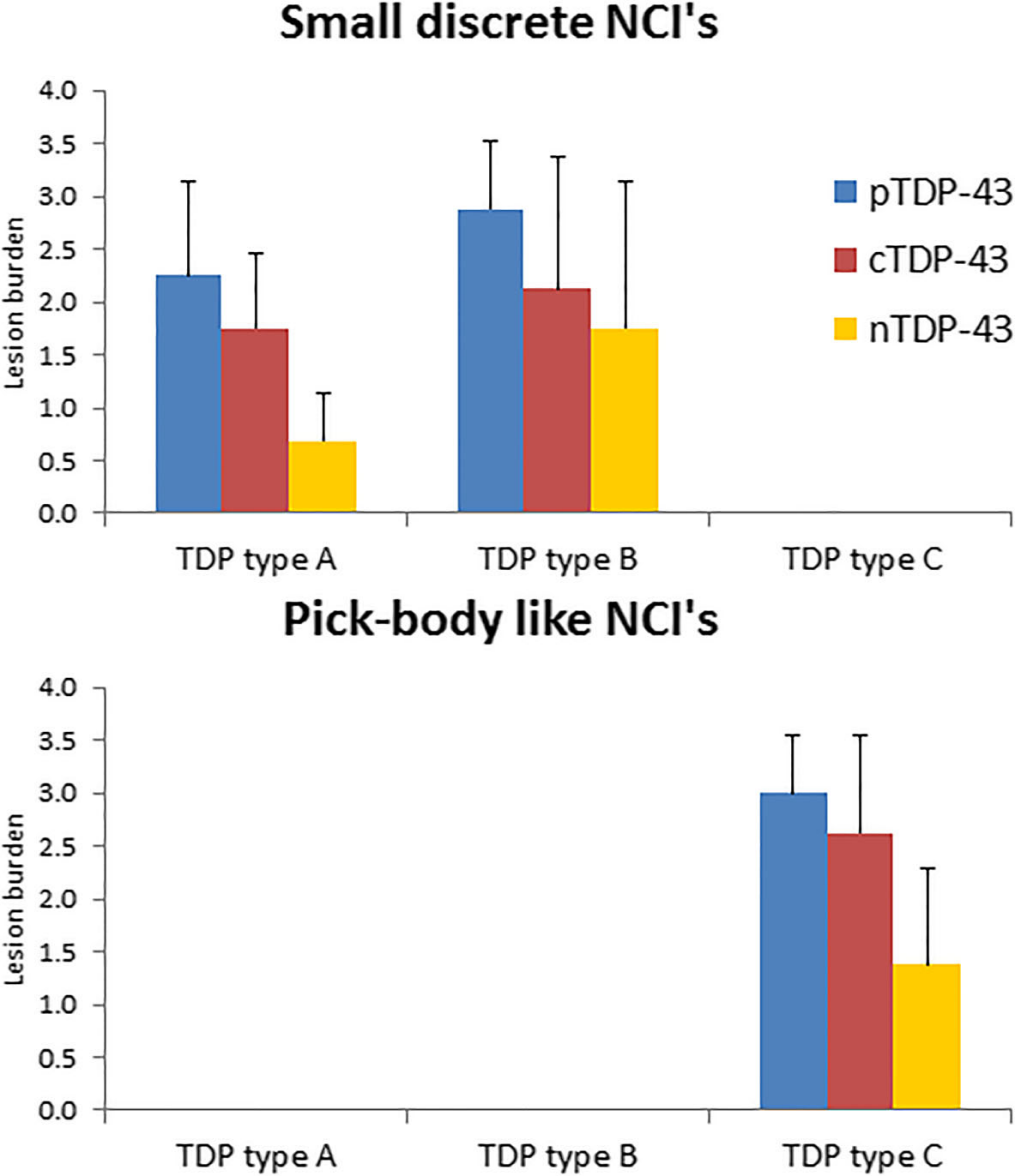
Histograms showing lesion burden detected using pTDP-43, cTDP-43 and nTDP-43 antibodies across FTLD-TDP types for the dentate nucleus of the hippocampus. Lesion burden is based on semi-quantitation. The boxes represent mean burden with whiskers representing standard deviation

In the dentate nucleus of the hippocampus (Fig. 6), the pTDP-43 antibody detected the highest lesion burden, followed by the cTDP-43 antibody and lastly the nTDP-43 antibody, for both small discrete NCIs (FTLD-TDP types A and B) (Figs. 3 & 5) and Pick-body like NCIs (FTLD-TDP type C) (Fig. 4).

### Demographics and pathological features by mutation

Demographic characteristics of the 16 FTLD-TDP type A and B cases by mutation status are shown in Table 2. The *C9ORF72*+ cases were the youngest at death. The clinical diagnoses, and β-amyloid and NFT distribution, were relatively similar between *GRN*+ and *GRN*- cases and between *C9ORF72*+ and *C9ORF72*- cases. Hippocampal sclerosis was present in all *GRN*+ and *GRN*- cases and 50% of *C9ORF72* + cases, but was not present in any of the *C9ORF72*- cases. In all instances the pTDP-43 antibody detected the greatest number of inclusions compared to the cTDP-43 and nTDP-43 antibodies. There was little evidence for striking differences in the burden of lesions detected with the pTDP-43, cTDP-43 and nTDP-43 antibodies by mutation status. The higher proportion of lesions detected with nTDP-43 vs cTDP-43 antibody in the entorhinal/CA1/subiculum in FTLD-TDP type B cases was similar for *C9ORF72*+ and *C9ORF72*- cases.

**Table 2:** Demographics and pathologic features of FTLD-TDP type A and type B cases split by mutation

Mutation	FTLD-TDP type A		FTLD-TDP type B	
	*GRN* + (*n* = 4)	*GRN* – (*N* = 4)	*C9*+ (*n* = 4)	*C9* – (*n* = 4)
Demographics				
Female Sex %	1 (25%)	0	3 (75%)	2 (50%)
Age at death	67 (60, 76)	74 (61, 78)	62 (55, 67)	68 (60, 79)
Clinical diagnosis				
bvFTD	2 (50%)	1 (25%)	2 (50%)	1 (25%)
PPA	1 (25%)	0	0	0
FTD-MND/ALS	0	1 (25%)	2 (50%)	3 (75%)
Other	1 (25%)	2 (50%)	0	0
Co-pathologies				
Hippocampal sclerosis	4 (100%)	4 (100%)	2 (50%)	0
Braak NFT stage	1 (0.0, 2.0)	1.0 (1.0, 1.5)	2.0 (2.0, 3.0)	1.5 (1.0, 3.0)
Thal Phases	0.5 (0.0, 1.0)	1.0 (0.0, 3.0)	1.0 (0.0. 2.0)	0.0 (0.0, 4.0)
Pathological results				
*ERC/CA1/Sub*				
Phosphorylated TDP-43 (pTDP-43)	2.5 (2.0–3.0)	1.5 (1.0, 3.0)	3.0 (1.0, 3.0)	2.0 (2.0, 3.0)
C-Terminal specie TDP-43 (cTDP-43)	2.0 (1.0–3.0)	1.0 (0.5–2.0)	0.0 (0.0, 1.0)	1.0 (0.0, 3.0)
Full length TDP-43 (nTDP-43)	1.0 (1.0, 1.0)	1.0 (0.5, 2.0)	1.5 (0.0, 2.0)	2.0 (1.0, 3.0)
*Dentate nucleus of hippo*				
Phosphorylated TDP-43 (pTDP-43)	2.5 (2.0, 3.0)	2.0 (1.0, 3.0)	3.0 (2.0, 3.0)	3.0 (2.0, 4.0)
C-Terminal specie TDP-43 (cTDP-43)	2.0 (1.0, 3.0)	1.5 (1.0, 2.0)	2.0 (1.0, 3.0)	2.0 (1.0, 4.0)
Full length TDP-43 (nTDP-43)	1.0 (0.0, 1.0)	0.75 (0.0, 1.0)	2.0 (0.0, 3.0)	1.0 (1.0, 4.0)

Data shown as median (range) and percentages

*bvFTD* Behavioral variant frontotemporal dementia, *FTD-MND/ALS* Frontotemporal dementia with motor neuron disease/amyotrophic lateral sclerosis, *PPA* Primary progressive aphasia, *GRN* progranulin, *C9* C9ORF72, *NFT* neurofibrillary tangle

### Associations of TDP-43 specie with lesion type and mutation status

**Fig. 7:**
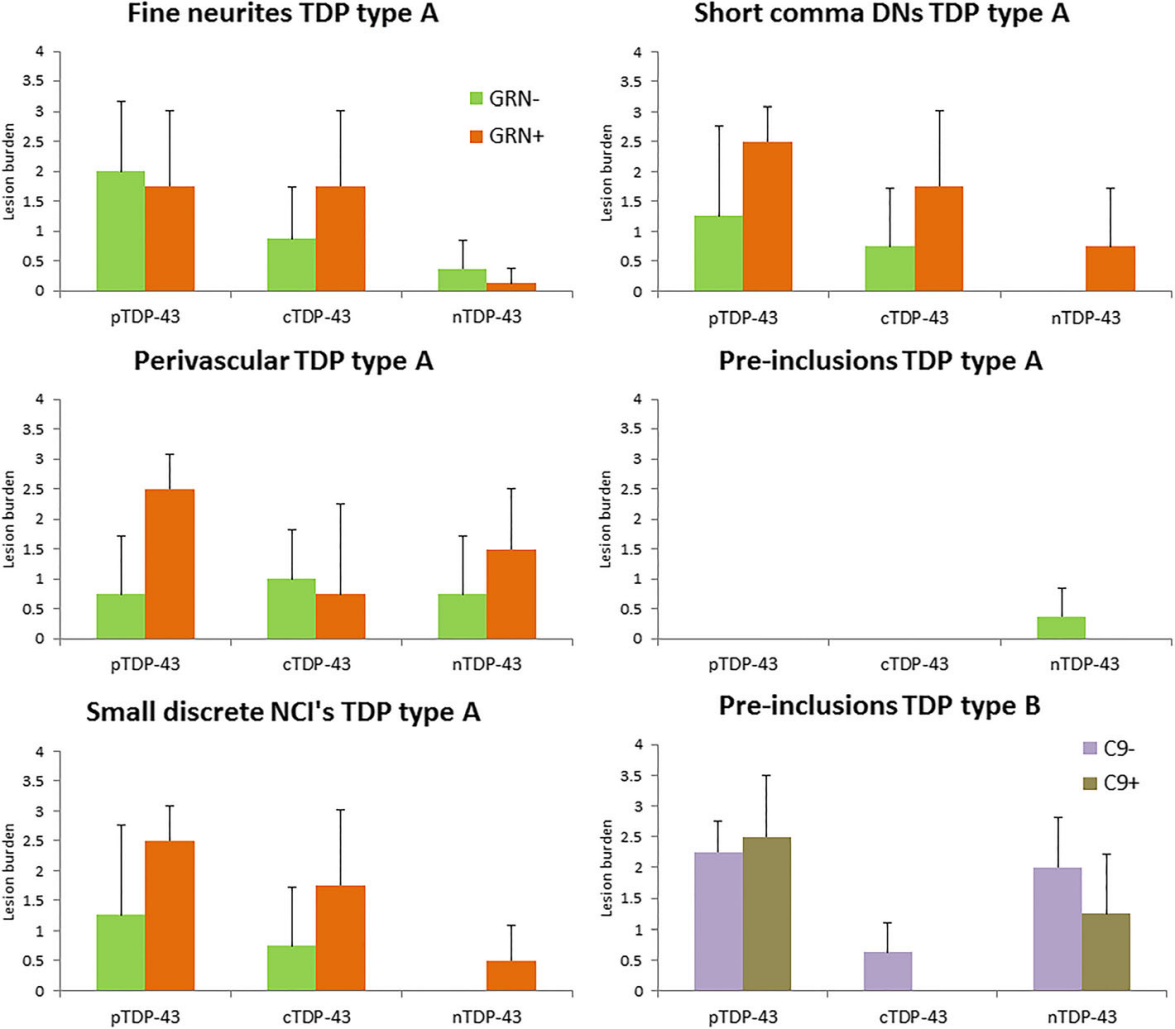
Histograms showing lesion burden detected using pTDP-43, cTDP-43 and nTDP-43 antibodies by mutation status for the entorhinal cortex/CA1/subiculum. Lesion burden is based on semi-quantitation. The boxes represent mean burden with whiskers representing standard deviation

In order to get a better understanding of whether mutation status may be playing a role in the relationships we observed between the different antibodies and lesion type, we compared pTDP-43, cTDP-43 and nTDP-43 antibody burden for each lesion type, by mutation status. Hence, we compared *GRN* status within FTLD-TDP type A cases, and *C9ORF72* status within FTLD-TDP type B cases, for each lesion type (Fig. 7).

First we describe our findings for FTLD-TDP type A associated lesions. We found that lesion burden was higher with the pTDP-43 antibody for GRN+ cases compared to GRN- cases for all lesions except for fine neurites where burden was about equal. We noted that lesion burden was higher for the cTDP-43 antibody in *GRN*+ cases compared to *GRN*- cases for small discrete NCI’s, DNs and fine neurites. We also observed that lesion burden was higher for the nTDP-43 antibody in *GRN*+ cases compared to *GRN*- cases for small discrete NCIs, DNs and perivascular inclusions. In fact, the *GRN*- cases had no nTDP-43 small discrete NCIs or DNs. Comparing across antibodies, there was a higher burden of lesions detected with the cTDP-43 antibody than the nTDp-43antibody in most instances, except for with perivascular inclusions.

For FTLD-TDP type B we investigated mutation status for pre-inclusions. We found that the *C9ORF72*+ cases had a lower burden of lesions for both nTDP-43 and cTDP-43 antibodies compared to *C9ORF72*- cases. In fact, we did not see any *C9ORF72*+ cases with pre-inclusions that were detected with the cTDP-43 antibody. We also found that the *C9ORF72*+ cases had a slightly higher burden of pre-inclusions that were detected with the pTDP-43 antibody compared to the *C9ORF72*- cases. Comparing across antibodies, we observed that our finding of higher lesion burden for the nTDP-43 antibody compared to cTDP-43 antibody was observed for both *C9ORF72*+ and *C9ORF72*- cases.

## Discussion

The results of this study demonstrate that phosphorylated TDP-43, C terminal specie and full length TDP-43 are all present in the inclusions in FTLD-TDP. Given that the characteristics of the different FTLD-TDP variants are dependent on different inclusion types, the results of this study support the notion that there are indeed differences in TDP-43 specie across the different FTLD-TDP variants. The findings improve our understanding of the relationship between TDP-43 specie and FTLD-TDP and may shed light on the cellular mechanisms resulting in lesion formation in the different FTLD-TDP types, with the findings suggesting different underlying mechanisms in FTLD-TDP type B. This knowledge may be helpful for the development of future treatments targeting TDP-43.

Previous studies have demonstrated that the inclusions found in FTLD-TDP consist of phosphorylated TDP-43 [1, 17, 33]. It has also been shown that the inclusions in FTLD-TDP consist mainly of C terminal fragments of TDP-43 [1, 33]. Therefore, one could infer that phosphorylated C terminal fragments are the key to inclusion formation and pathogenicity. However, others have shown that phosphorylation of TDP-43 is not necessary for inclusion formation and that lesions in FTLD-TDP likely consist of phosphorylated and unphosphorylated C terminal specie of TDP-43 [46]. The cTDP-43 antibody utilized in our study detects both phosphorylated and unphosphorylated C terminal fragments [46]. While we cannot determine the degree of phosphorylation in our C terminal fragments, we did find that the burden of inclusions detected with the pTDP-43 antibody was greater than with the cTDP-43 antibody, consistent with pTDP-43 antibody detecting both phosphorylated C-terminal and phosphorylated full-length TDP-43. Full-length TDP-43, therefore appears, to be an important component of inclusions in FTLD-TDP. Indeed, it has been demonstrated that TDP-43 aggregation occurs when full length (endogenous) TDP-43 is prevented from exiting the nucleus or from entering the nucleus [42].

The burden of inclusions detected with the cTDP-43 antibody was higher than the burden of inclusions detected with the nTDP-43 antibody for most inclusion types, including fine neurites of the hippocampus, perivascular inclusions, small discrete NCIs and both short/comma and long thick DNs. These inclusion types, therefore, appear to consist mainly of C terminal TDP-43 and concur with previous studies that have also noted a predominance of C terminal TDP-43 in FTLD-TDP inclusions [1, 21]. A novel finding from our study was that the ratio of C terminal fragments to full-length TDP-43 was reversed in the pre-inclusions identified in FTLD-TDP type B, with a higher burden of inclusions identified with the nTDP-43 antibody. This is intriguing given that FTLD-TDP type B is typically associated with motor neuron disease/amyotrophic lateral sclerosis [25]. This would suggest that the mechanism by which TDP-43 immunoreactive pre-inclusions are formed in cases with motor neuron disease may differ from mechanisms involved with the formation of lesions in FTLD-TDP type A and type C. With-that-said, we did observe pre-inclusions in two FTLD-TDP type A cases. Interestingly, in both cases the pre-inclusions were not detected with the pTDP-43 or cTDP-43 antibodies suggesting the possibility that these inclusions consist of unphosphorylated full length TDP-43. In one of these two cases, there was a mutation in the *C9ORF72* gene and the clinical diagnosis was FTD-MND. This case, however, was classified as FTLD-TDP type A given the presence of fine neuritis in the CA1 region of the hippocampus [18, 25]. It should be pointed out that other features of FTLD-TDP type A such s NIIs were not present in this case. Given the uncertainty in classification of this case, it is unclear whether this finding is unique to FTLD-TDP type B, or rather a feature of pre-inclusions, independent of FTLD-TDP type. Previous studies that have assessed C terminal fragments and full-length TDP-43 across FTLD-TDP types did not assess pre-inclusions [1, 21] and hence we are unable to compare results.

We also noted that perivascular inclusions in FTLD-TDP type A cases were slightly more likely to be detected with the nTDP-43 antibody compared to the cTDP-43 antibody. This trend was not as striking as for pre-inclusions, but was very different from the findings across the other inclusions identified in FTLD-TDP type A. In fact there was a case where no perivascular inclusions were detected with the cTDP-43 antibody while they were detected with the nTDP-43 antibody. In this case the pTDP-43 antibody also detected the perivascular inclusions. This finding would support the notion that cellular mechanisms resulting in lesion formation may differ within FTLD-TDP type A. Once again previous studies that have assessed C terminal fragments and full-length TDP-43 across FTLD-TDP types have not assessed perivascular inclusions [1, 15]. Similarly, for FTLD-TDP type B, there may be differences in how pre-inclusions in the cortex are formed versus how small discrete NCI’s are formed in the dentate nucleus of the hippocampus. Hence, although it has been shown that C terminal fragments of TDP-43 are indistinguishable between regions [39], the findings here support another study showing differences between regions when comparing C terminal specie and full length TDP-43 [21].

When we studied NIIs we observed that the highest lesion burden occurred with the pTDP-43 antibody of which only a subset was identified with the C terminal antibody; none were detected with the nTDP-43 antibody. This finding is different from that reported in one previous study where the investigators found evidence for both C terminal specie and full length TDP-43 to be a component of NIIs [21]. Our findings suggest that NIIs consist of C terminal specie TDP-43 that is predominantly phosphorylated but not full length TDP-43. Given that the nuclear localization signal is within the N-terminal half of the protein [45] it is unlikely that TDP-43 is cleaved and phosphorylated in the cytoplasm and then C terminal fragments are shuttled back into the nucleus. Hence, cleavage is most likely occurring inside the nucleus. It has been reported in one study that C-terminal fragments of TDP-43 do not sequester full length TDP-43 [46]. If correct, a likely explanation for the absence of full length TDP-43 in NII’s is that once NIIs are formed in the nucleus, from the cleavage of the full length TDP-43, full length cytoplasmic TDP-43 is blocked from being shuttled inside the nucleus. This would be expected given that TDP-43 pathology interferes with nuclear pore complexes and transport of TDP-43 into the nucleus [11]. On-the-other hand, studies have reported that C terminal fragments can sequester full length TDP-43 [8, 34] which if correct would explain the absence of full length TDP-43 in NII’s.

We should also point out that when we identified inclusions with the nTDP-43 antibody we also noted there was an absence of normal full-length TDP-43 from the nucleus of the associated cells. This implies that full length TDP-43 that is sequestered into the inclusion was unable to shuttle back into the nucleus. Whether this is due to the phosphorylation of the full length TDP-43, given that the pTDP-43 antibody detected the inclusions, or whether it is due to another type of post translational modification, remains unknown.

Our analysis by FTLD mutations showed that our general findings were not driven by mutation status in any of the FTLD-TDP types. This is not surprising as animal models have not found any evidence for *GRN* mutations to be associated with TDP-43 fragmentation [15, 31] and down regulation of GRN in zebrafish. We did, however, find a suggestion for differences by mutation. In general, the *GRN*+ cases showed a higher burden of TDP-43 inclusions, independent of the specific antibody, with only a few exceptions. The only demographic difference between the *GRN*+ and *GRN*- cases was a younger age in the *GRN*+ cases. Previous studies have shown that *GRN* mutations are associated with a more aggressive disease [41], and our findings may imply that this could be due to a higher pathological TDP-43 burden [23, 29]. We did not find this association with the *C9ORF72* mutation. Interestingly, we noted that the *C9ORF72*+ cases had a slightly higher burden of pre-inclusions that were detected with the pTDP-43 antibody compared to the *C9ORF72*- cases while the C9ORF72- cases had a higher burden of inclusions with the cTDP-43 and nTDP-43 antibodies, suggesting that there were phosphorylated pre-inclusions in the *C9ORF72*+ cases that must not have been detected with either the cTDP-43 or nTDP-43 antibodies. This is not necessarily surprising given that other C terminal fragments have been reported in FTLD-TDP [20, 34, 40, 44].

## Conclusion

A strength of this study was the fact that we looked at eight different inclusions and the fact that all patients underwent genetic screening for a *GRN* or *C9ORF72* mutation. Despite the fact that the number of cases assessed was not large enough to allow for statistical testing, we have identified some interesting trends in the data, particularly in the pre-inclusions in FTLD-TDP type B, which warrant further investigation. Furthermore, the findings have relevance in the interpretation of studies from different laboratories as it is clear that some inclusions may not be seen depending on what kind of anti-TDP-43 antibody is being used for detection.

## Data Availability

The datasets generated and analyzed for the current study is available from the corresponding author on reasonable request**.**
